# Condemnation of 606

**Published:** 1914-08

**Authors:** 


**Condemnation of 606.** Gaucher, Gaz. des Prat., April 1, 1914, considers 606 dangerous to the nervous system and even fatal at times and claims that it does not cure syphilis although it temporarily heals ulcerative lesions of the skin and mucous membranes. He bases his claims on observation and statements of others and says that Fournier, in alluding to the publication of the former in 1910, gave him credit for being the sole clairvoyant in the matter.

